# The Treatment Effect of an ACE-Inhibitor Based Regimen with Perindopril in Relation to Beta-Blocker use in 29,463 Patients with Vascular Disease: a Combined Analysis of Individual Data of ADVANCE, EUROPA and PROGRESS Trials

**DOI:** 10.1007/s10557-017-6747-9

**Published:** 2017-08-31

**Authors:** J. J. Brugts, M. Bertrand, W. Remme, R. Ferrari, K. Fox, S. MacMahon, J. Chalmers, M. L. Simoons, E. Boersma

**Affiliations:** 1 Department of Cardiology, Erasmus University Medical Center, Thoraxcenter, ‘s-Gravendijkwal 230, 3015 CE Rotterdam, The Netherlands; 2 Institut CŒUR Poumon, Lille, France; 3 STICARES Cardiovascular Research Institute, Rhoon, The Netherlands; 4 Centro Cardiologico Universitario University of Ferrara, Italy and Maria Cecilia Hospital, GVM Care & Research, E.S. Health Science Foundation, Cotignola, Ravenna Italy; 5 NHLI, Imperial College and ICMS, Royal Brompton Hospital, London, UK; 6 The George Institute for Global Health, The Royal Prince Alfred Hospital and the University of Sydney, Sydney, New South Wales Australia

**Keywords:** ACE-inhibitor, Perindopril, Beta-blocker, Hypertension, Prevention, Vascular disease

## Abstract

**Introduction:**

In everyday practice, angiotensin converting enzyme inhibitors and beta-blockers are cornerstone treatments in patients with (cardio-)vascular disease. Clear data that evaluate the effects of the combination of these agents on morbidity and mortality are lacking.

**Methods:**

In this retrospective pooled analysis of three large perindopril outcome trials (ADVANCE, EUROPA, PROGRESS), clinical outcomes were evaluated in 29,463 patients with vascular disease. Multivariate Cox regression analyses were performed in patients randomized to a perindopril-based regimen or placebo (treatment effect), and data were stratified according to background beta-blocker treatment. The primary endpoint was a composite of cardiovascular mortality, non-fatal myocardial infarction, and stroke.

**Results:**

The cumulative incidence of the primary endpoint over mean follow-up of 4.0 years (Sd 1.0) was significantly lower in the beta-blocker/perindopril group (9.6%; 545/5700 patients) as compared to beta-blocker/placebo (11.8%; 676/5718 patients) (*p* < 0.01). Adding perindopril to existing beta-blocker treatment reduced the relative risk of the primary endpoint by 20% (hazard ratio (HR) 0.80; 95% confidence interval (CI) 0.71–0.90), non-fatal myocardial infarction by 23% (HR 0.77; 95% CI 0.65–0.91), and all-cause mortality by 22% (HR 0.78; 95% CI 0.68–0.88) as compared to placebo. Significant treatment benefit was not observed for stroke (HR 0.93; 95% CI 0.75–1.15). Significance was maintained for the primary endpoint and cardiovascular endpoints when data were further stratified by baseline hypertension. However, the mortality benefit was only observed in patients with hypertension with background beta-blocker use.

**Conclusions:**

These data suggest that the beneficial cardioprotective effects of perindopril treatment are additive to the background beta-blockers use.

## Introduction

In order to reduce morbidity and mortality, guidelines for hypertension, coronary artery disease, and heart failure recommend treatment with beta-blockers and angiotensin-converting enzyme (ACE) inhibitors [1–3]. Beta-blockers are generally used to decrease blood pressure, manage arrhythmias (i.e., prevent sudden cardiac death), and treat angina pectoris and heart failure. Angiotensin-converting enzyme inhibitors are used to reduce blood pressure, treat congestive heart failure, and to provide cardioprotection in patients with acute coronary syndromes [2].

Consistent with these recommendations, a wide range of patients with cardiovascular diseases are treated daily with beta-blockers and ACE-inhibitors [4]. Large cross-sectional population studies have found, for instance, that beta-blockers and ACE-inhibitors are prescribed in 86 and 65% of patients with coronary disease and in 93 and 71% of patients with heart failure, respectively [5, 6]. Data have also shown that beta-blocker/ACE-inhibitor combinations are the most frequently prescribed antihypertensive combination in real-life clinical practice (15–22% of hypertensive patients) [4].

Perindopril is a widely prescribed ACE-inhibitor that is supported by extensive pharmacokinetic, pharmacodynamic, blood pressure, and outcome studies [7–12]. A previous meta-analysis of the large EUROPA, ADVANCE, and PROGRESS trials, which included a wide range of patients with vascular disease, showed that perindopril-based regimens improved survival and reduced the risk of having a major cardiovascular event [13].

Few data that look specifically at the effect of a beta-blocker/perindopril combination and its interaction on cardiovascular events have been published [14]. A subanalysis of the EUROPA trial (*N* = 7534) has shown that beta-blocker/perindopril treatment, when compared to beta-blocker/placebo, significantly reduced the relative risk of cardiovascular death/non-fatal myocardial infarction (MI)/resuscitated cardiac arrest (−24%; *p* = 0.002), MI (−28%; *p* = 0.001), and hospitalization for heart failure (−45%; *p* = 0.025) [14]. As the ADVANCE and PROGRESS trials also included a large number of patients taking a beta-blocker as a background treatment [10, 12], a retrospective pooled analysis of the EUROPA, ADVANCE, and PROGRESS trials would offer outcome data for a large cohort of patients with a wide range of cardiovascular risk profiles. The objective of this analysis was, therefore, to assess the benefit of perindopril treatment in patients already taking beta-blockers and to study its additive or synergistic effects in more detail. In particular, benefits of beta-blocker/perindopril treatment were looked at in hypertensive and non-hypertensive patients.

## Methods

Methods were similar to those published by Brugts et al. [13]. Individual data from the ADVANCE, EUROPA, and PROGRESS studies were pooled. As previously described, the rationale for combining these trials is based on the fact that all three trials studied the same agent, perindopril; we provided the opportunity to include individual data in this combined analysis, which made important subgroup analyses possible at the patient level with individual data access. The types of patients included in these studies were different in their primary diagnoses, but since atherosclerosis and vascular disease are not restricted to a single vascular bed, we conclude that these patients are at least homogenous in having vascular disease or being at a high risk of vascular disease. The combined data set consisted of 29,463 patients, who were followed for an average of 4 years [13].

In all three trials, all patients were treated with a perindopril-based regimen during the run-in period. Patients were then randomized to a perindopril-based treatment or placebo for 4 years. In PROGRESS, patients had a history of stroke or transient ischemic attack (TIA) and were randomized to treatment with perindopril 4 mg ± indapamide 2.5 mg or placebo [10]. In ADVANCE, patients had type 2 diabetes and were randomized to combination treatment with perindopril 2–4 mg/indapamide 0.625–1.25 mg or placebo [12]. In EUROPA, patients had stable coronary heart disease and were randomized to treatment with perindopril 8 mg or placebo [11]. In all three trials, background beta-blocker use was recorded. Dose and type of beta-blocker use was not available; however, co-medication use was comprehensively noted.

### Outcomes

In this analysis, the primary endpoint was a composite of cardiovascular mortality, non-fatal MI, and stroke (identical in all three trials). Secondary endpoints were all-cause mortality, cardiovascular mortality, non-fatal MI, stroke, and two composite endpoints (cardiovascular mortality/non-fatal MI and cardiovascular mortality/non-fatal MI/revascularization). Endpoints were assessed throughout the study period. Study-specific definitions were used as previously described [13].

### Statistics

The randomization effect was preserved in all analyses by studying the treatment effect of perindopril only. Data were stratified according to beta-blocker therapy. Multivariate Cox regression analyses were performed to calculate hazard ratios (HR) and 95% confidence intervals (CIs). Adjustments (full model) were made for baseline age, sex, hypertension, diabetes mellitus, smoking, history of MI, history of percutaneous coronary intervention/coronary artery bypass grafting, history of stroke/TIA, and co-medication use (antiplatelet agents, lipid-lowering agents, calcium antagonists, diuretics), indapamide use, and active treatment (perindopril) dosage (by trial). Additional adjustments for baseline risk, tests for heterogeneity in treatment effects, and tests for heterogeneity among trials were performed as previously described [13]. Hazard ratios and 95% CIs are presented with corresponding two-sided *p* values. Survival analyses were performed for all treatment groups, and differences were evaluated using log rank (Mantel-Cox) tests. In all analyses, a *p* value ≤ 0.05 was considered significant.

Data were further stratified according to the presence or absence of hypertension at baseline. Hypertension was defined as a systolic blood pressure (SBP)/diastolic blood pressure (DBP) ≥ 160/95 mmHg or use of antihypertensive medication according to the definition used in EUROPA [11] and previous analyses [13]. As the definition of hypertension has changed since the EUROPA trial was performed, the primary endpoint was also assessed in additional post hoc subgroup analysis using a cut-off for hypertension of SBP/DBP ≥ 140/90 mmHg or the use of antihypertensive medication.

## Results

The pooling of data from ADVANCE, EUROPA, and PROGRESS resulted in a study cohort of 29,463 patients at high cardiovascular risk.

During the 4-week run-in period, during which all patients received perindopril, SBP/DBP decreased by a mean of −7.8 ± 16.0/−3.6 ± 9.0 mmHg in patients taking beta-blockers (*n* = 11,418) and by a mean of −8.6 ± 16.0/−4.0 ± 9.0 mmHg in patients not taking beta-blockers (*n* = 18,045). Differences between groups were not significant in BP reduction.

Among the 11,418 patients (38.8%) taking a background beta-blocker, 5700 patients (49.9%) to the perindopril-based regimen (beta-blocker/perindopril group) and 5718 patients (50.1%) had been randomized to placebo (beta-blocker/placebo group). Among the 18,045 patients (61.2%) not taking a background beta-blocker, 9030 patients (50.0%) to perindopril (no beta-blocker/perindopril group) and 9015 patients (50.0%) had been randomized to placebo (no beta-blocker/placebo group). Patient characteristics for the total study population and strata are presented in Tables 1 and 2.

**Table 1:** Baseline characteristics in combined study population (*n* = 29,463)

	ADVANCE (*n* = 11,140)	EUROPA (*n* = 12,218)	PROGRESS (*n* = 6105)	TOTAL (*n* = 29,463)
Characteristics				
Age (years), mean (SD)	66 (6)	60 (9)	64 (10)	63 (9)
Female, %	42.5	14.6	30.3	28.4
Previous MI, %	12.0	64.8	7.0	32.8
Previous PCI/CABG, %	8.5	54.9	2.7	26.6
Previous CVA/TIA, %	12.9	3.4	99.9^a^	27.0
Current smokers, %	15.1	15.2	20.0	16.2
Diabetes, %	100.0	12.3	12.5	45.5
Hypertension, %	68.7	27.1	47.8	54.1
Hypercholesterolemia, %	58.9	63.3	–	61.2^b^
Systolic blood pressure (mm Hg)	145 (21)	137 (15)	147 (9)	142 (19)
Diastolic blood pressure (mm Hg)	81 (11)	82 (8)	86 (11)	82 (10)
Medications				
Antiplatelet agents, %	46.7	92.3	72.3	70.8
Beta-blockers, %	24.5	61.7	17.0	38.8
Lipid-lowering agents, %	35.3	55.9	14.1	39.5
Calcium antagonists, %	30.8	31.4	39.9	33.3
Diuretics use, %^c^	9.2	23.7	11.5	15.1

Summary statistics for continuous variables are presented as mean (SD). Categorical data are summarized as percentages. The definition of hypertension was unified in all trials, with EUROPA as template, as a blood pressure of ≥ 160/95 mmHg or use of antihypertensives

*CABG* coronary artery bypass grafting, *CVA* cerebrovascular disease, *MI* myocardial infarction, *PAD* peripheral arterial disease, *PCI* percutaneous coronary intervention, *SD* standard deviation, *TIA* transient ischemic attack

^a^PROGRESS, percentage was 61.2% (14294) based on 23,358 patients (ADVANCE and EUROPA)

^b^Hypercholesterolaemia data were not present in PROGRESS

^c^Diuretics use other than indapamide study medication

Reproduced with permission from Brugts et al. (2009)

**Table 2:** Baseline characteristics of treatment groups and beta-blocker strata in the combined study population (*n* = 29,463)

	Placebo		Perindopril-based regimen	
	No beta-blocker (*n* = 9015)	Beta-blocker (*n* = 5718)	No beta-blocker (*n* = 9030)	Beta-blocker (*n* = 5700)
Characteristics				
Age (years), mean (SD)	64 (8)	62 (9)	64 (8)	61 (9)
Female, %	31.8	23.2	31.8	22.7
Previous MI, %	20.5	51.9	20.8	52.2
Previous PCI/CABG, %	18.6	39.7	18.1	39.4
Previous CVA/TIA, %	34.8	14.5	35.2	14.1
Current smokers, %	17.2	15.1	17.3	13.8
Diabetes, %	52.9	34.1	53.6	32.4
Hypertension, %	55.7	51.8	56.2	50.5
Hypercholesterolemia, %^a^	57.1	66.3	57.3	66.0
Systolic BP, mean (SD)	143 (19)	141 (19)	143 (19)	140 (19)
Diastolic BP, mean (SD)	82 (10)	82 (9)	82 (10)	82 (9)
Medications				
Antiplatelet agents, %	62.3	84.4	62.3	84.1
Beta-blocker agents, %	100	0	100	0
Lipid-lowering agents, %	31.7	52.5	31.2	51.8
Calcium antagonists, %	35.2	30.6	35.2	30.1
Diuretics, %^b^	13.6	16.1	14.0	15.2

Descriptive statistics are presented as mean (SD) for continuous variables and as percentages for categorical variables. Hypertension was defined according to the EUROPA definition (blood pressure ≥ 160/95 mmHg or use of antihypertensives)

*CABG* coronary artery bypass grafting, *BP* blood pressure in mmHg, *CVA* cerebrovascular disease, *MI* myocardial infarction, *PAD* peripheral arterial disease, *PCI* percutaneous coronary intervention, *SD* standard deviation, *TIA* transient ischemic attack

^a^Hypercholesterolemia data were not reported in PROGRESS. Percentages were based on a total of 23,358 patients (ADVANCE + EUROPA)

^b^Diuretics use excluding indapamide study medication

**Fig. 1:**
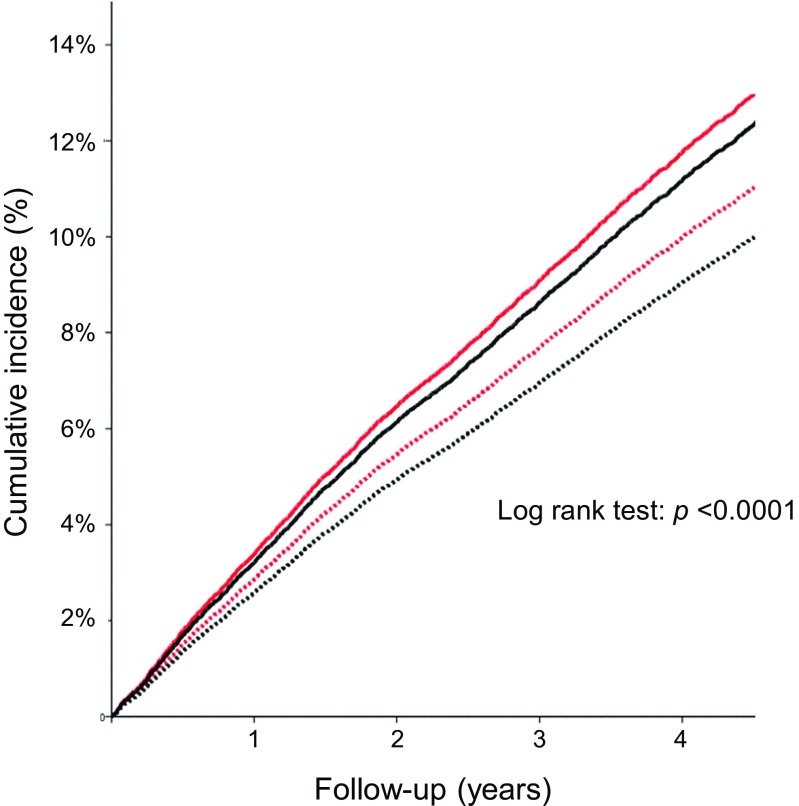
Cumulative incidence survival function of the primary endpoint in 29,463 patients by Cox regression analysis. The primary endpoint was defined as the composite of cardiovascular mortality, non-fatal myocardial infarction, and stroke. Subgroups were defined as no beta-blocker/placebo (*n* = 9015; solid red line), beta-blocker/placebo (*n* = 5718; solid black line), no beta-blocker/perindopril (*n* = 9030; dotted red line), and beta-blocker/perindopril (*n* = 5700; dotted black line). Cumulative incidence in percentages (%) and follow-up duration in years are represented on the *y*-axis and *x*-axis, respectively

The primary endpoint (cardiovascular mortality/non-fatal MI/stroke) occurred in 1221 of the 11,418 patients in the beta-blocker stratum (10.7%; HR 0.80; 95% CI 0.71–0.90): 676 patients in the beta-blocker/placebo group (11.8%) and 545 patients in the beta-blocker/perindopril group (9.6%). The primary endpoint also occurred in 2057 of the 18,045 patients in the no beta-blocker stratum (11.4%; HR 0.83; 95% CI 0.76–0.91): 1112 patients in the no beta-blocker/placebo group (12.3%) and 945 patients in the no beta-blocker/perindopril group (10.5%). The cumulative incidence of patients who reached the primary endpoint over the follow-up period was lowest in the beta-blocker/perindopril group (Fig. 1).

### Subanalyses in Beta-Blocker Strata

**Fig. 2:**
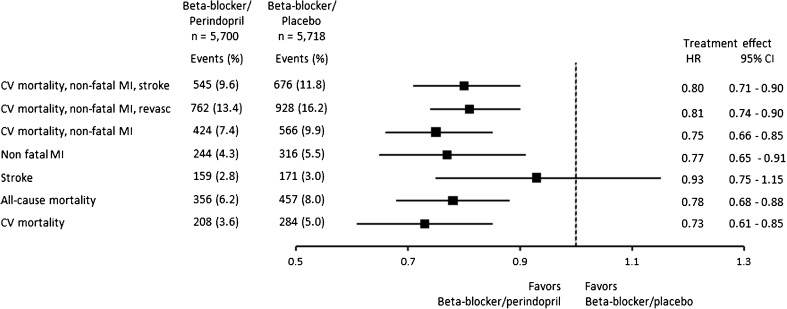
Treatment effect of perindopril-based regimen in beta-blocker stratum: Forest plot. A Cox regression multivariate analysis was performed to calculate HRs and 95% CIs with adjustments for full model. The primary endpoint was the composite endpoint of cardiovascular mortality, non-fatal MI, and stroke. Among the 11,418 patients taking a beta-blocker, 5700 were randomized to a perindopril-based regimen and 5718 to placebo. *P* interaction was significant for all-cause mortality and CV mortality; all other *p* interactions are ns. *CI*, confidence interval, *CV* cardiovascular, *HR* hazard ratio, *MI* myocardial infarction, *revasc* revascularization, *TIA* transient ischemic attack

**Fig. 3:**
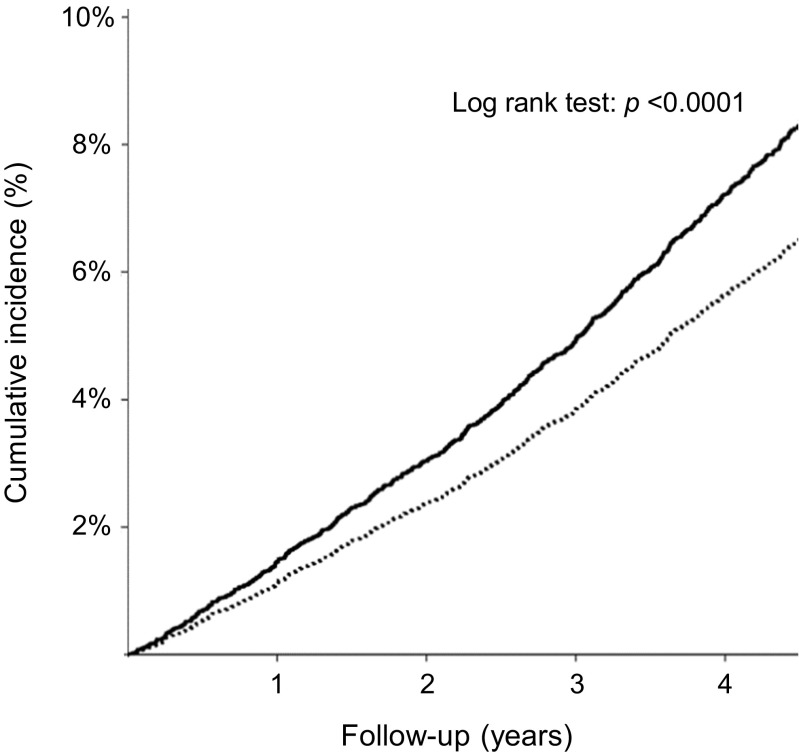
Cumulative incidence of all-cause mortality in patients randomized to perindopril-based regimen and placebo in the beta-blocker stratum. Subgroups were defined as beta-blocker/placebo (*n* = 5718; solid black line) and beta-blocker/perindopril (*n* = 5700; dotted black line). Cumulative incidence in percentages (%) and follow-up duration in years are represented on the *y*-axis and *x*-axis, respectively

Secondary endpoints for the beta-blocker stratum (*n* = 11,418) are presented in Fig. 2. Perindopril treatment was associated with decreased risk of cardiovascular mortality/non-fatal MI/revascularization (HR 0.81; 95% CI 0.74–0.90), cardiovascular mortality/non-fatal MI (HR 0.75; 95% CI 0.66–0.85), non-fatal MI (HR 0.77; 95% CI 0.65–0.91), all-cause mortality (HR 0.78; 95% CI 0.68–0.88), and cardiovascular mortality (HR 0.73; 95% CI 0.61–0.85) compared with placebo (Fig. 2). Treatment effect was not significant for stroke (HR 0.93; 95% CI 0.75–1.15). The *p* value for treatment interaction of beta-blocker use was significant for all-cause mortality and cardiovascular mortality (full data shown in Tables 3 and 4). Cumulative incidence of all-cause mortality throughout the follow-up period was significantly lower (*p* < 0.01) in the beta-blocker/perindopril group than in the beta-blocker/placebo group (Fig. 3).

**Table 3:** Overview of outcome data (treatment effect perindopril vs placebo) in strata of beta-blocker and no-beta-blocker use for all endpoints

Endpoint	No beta-blocker (*n* = 18,045)			Beta-blocker use (*n* = 11,418)			Interaction
	Events	HR	95% CI	Events	HR	95% CI	*p* int
Primary endpoint	2057 (11.4)	0.83	0.76–0.91	1221 (10.7)	0.80	0.71–0.90	0.63
CV mortality, non-fatal MI	1289 (7.1)	0.88	0.79–0.98	990 (8.7)	0.75	0.66–0.85	0.09
Non-fatal MI	540 (3.0)	0.84	0.71–0.99	560 (4.9)	0.77	0.65–0.91	0.48
Stroke	1030 (5.7)	0.79	0.70–0.90	330 (2.9)	0.93	0.75–1.15	0.19
All-cause mortality	1486 (8.2)	0.96	0.88–1.06	813 (7.1)	0.78	0.68–0.88	0.02
CV mortality	819 (4.5)	0.93	0.83–1.07	492 (4.3)	0.73	0.61–0.85	0.04

A Cox regression multivariate analysis was performed to calculate HRs and 95% CIs with adjustments for full model. The primary endpoint was the composite endpoint of cardiovascular mortality, non-fatal MI, and stroke. Among the 18,045 patients without background beta-blocker use, 9030 were randomized to a perindopril-based regimen and 9015 to placebo. Among the 11,418 patients taking a beta-blocker, 5700 were randomized to a perindopril-based regimen and 5718 to placebo

*CI* confidence interval, *CV* cardiovascular, *HR* hazard ratio, *MI* myocardial infarction

**Table 4:** Full data in beta-blocker and no beta-blocker strata for baseline hypertension analysis

		No beta-blocker use (*n* = 18,045)				Beta-blocker use (*n* = 11,418)				Interaction
		Events	%	HR	95% CI	Events	%	HR	95% CI	*p* value
Primary endpoint	No hypertension	741	9.3	0.85	0.74–0.98	518	9.3	0.84	0.71–1.00	0.86
	Hypertension	1316	13.0	0.82	0.74–0.92	703	12.0	0.77	0.66–0.89	0.33
CV mortality, non-fatal MI	No hypertension	501	6.3	0.81	0.68–0.97	788	7.8	0.92	0.79–1.11	0.05
	Hypertension	460	8.2	0.82	0.68–0.98	530	9.1	0.68	0.58–0.82	0.48
Non-fatal MI	No hypertension	258	3.2	0.81	0.63–1.03	292	5.2	0.79	0.62–0.99	0.70
	Hypertension	282	2.8	0.88	0.70–1.11	268	4.6	0.74	0.58–0.94	0.46
Stroke	No hypertension	310	3.9	0.91	0.72–1.13	86	1.5	0.99	0.65–1.52	0.56
	Hypertension	720	7.1	0.75	0.65–0.87	244	4.2	0.90	0.70–1.16	0.26
All-cause mortality	No hypertension	546	6.9	0.94	0.79–1.10	326	5.8	0.93	0.74–1.15	0.90
	Hypertension	940	9.3	0.98	0.87–1.12	487	8.3	0.68	0.57–0.82	0.01
CV mortality	No hypertension	264	3.3	0.84	0.66–1.07	191	3.4	0.87	0.66–1.16	0.72
	Hypertension	555	5.5	0.97	0.83–1.17	301	5.2	0.65	0.51–0.82	0.01

Among patients with no background beta-blocker use (*n* = 18,045), 7944 had no history of hypertension and 10,101 had hypertension at baseline. Among patients with beta-blocker use (*n* = 11,418), 5580 had no history of hypertension at baseline and 5838 had hypertension at baseline. A Cox regression multivariate analysis was performed to calculate HRs and 95% CIs with adjustments for full model. The primary endpoint was the composite endpoint of cardiovascular mortality, non-fatal MI, and stroke. Results for other endpoints are similar

*CI* confidence interval, *CV* cardiovascular, *HR* hazard ratio, *MI* myocardial infarction

**Fig. 4:**
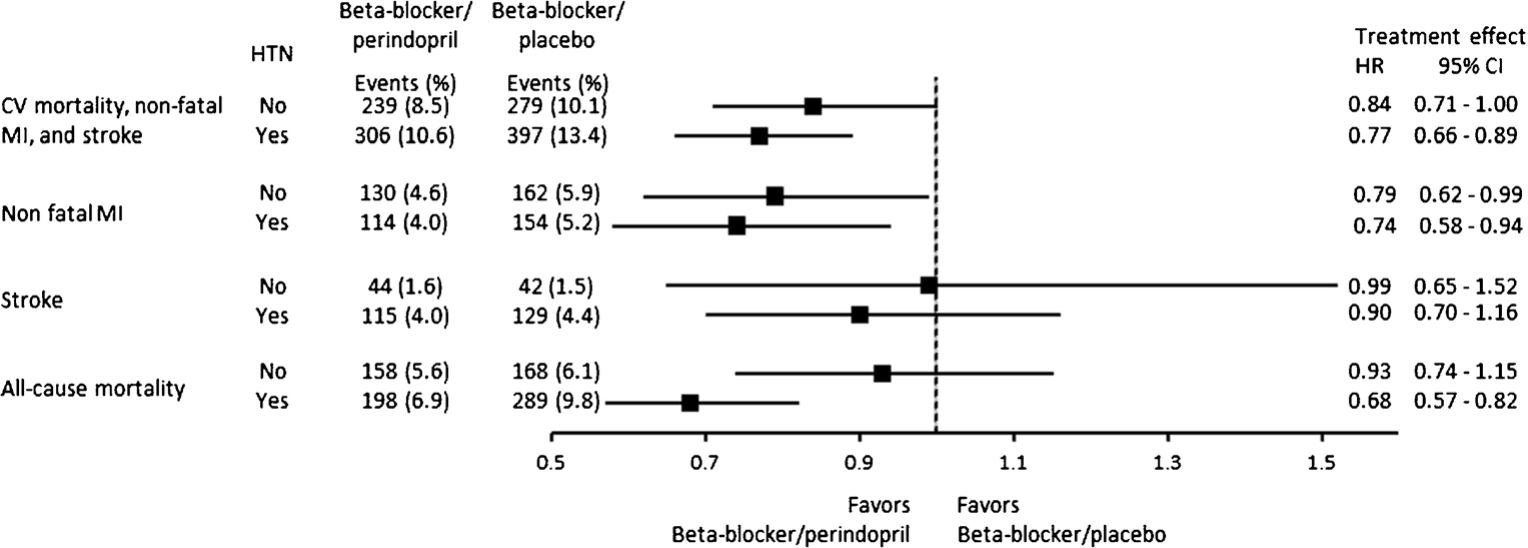
Treatment effect of ACE-inhibitor-based regimen with perindopril in the stratum of beta-blocker use according to baseline hypertension: Forest plot. Subanalyses were performed according to baseline hypertension. Of the 5700 patients in the beta-blocker/perindopril group, 2876 patients had HTN (50.5%). Of the 5718 patients in the beta-blocker/placebo, 2962 patients had HTN (51.8%). A Cox regression multivariate analysis was performed to calculate HRs and 95% CIs with adjustments for full model. *CI* confidence interval, *CV* cardiovascular, *HR* hazard ratio, *HTN* hypertension, *MI* myocardial infarction, *ns* not significant. *P* for interaction all-cause mortality 0.01; all other *p* interaction terms ns

Patients were further stratified according to baseline hypertension. Results for the beta-blocker stratum (*n* = 11,418) are presented in Fig. 4. In the subgroup of hypertensive patients (*n* = 5838) using beta-blockers, perindopril treatment was associated with significant risk reduction compared to placebo for the endpoints of cardiovascular mortality/non-fatal MI/stroke (HR 0.77; 95% CI 0.66–0.89; *p* = 0.001), non-fatal MI (HR 0.74; 95% CI 0.58–0.94; *p* = 0.02), and all-cause mortality (HR 0.68; 95% CI 0.57–0.82; *p* = 0.001). Risk reduction was not significant for stroke (HR 0.90; 95% CI 0.70–1.16; *p* = 0.4). In the subgroup of non-hypertensive patients (*n* = 5580), perindopril significantly reduced cardiovascular mortality/non-fatal MI/stroke (HR 0.84; 95% CI 0.71–1.00; *p* = 0.04), and treatment benefit was consistent also without beta-blocker use. Most surprisingly, the beneficial effect on all-cause mortality was lost in non-hypertensive patients. Significant interaction between the beneficial effect of beta-blocker and perindopril was found for all-cause mortality in hypertensive patients and not in non-hypertensive patients (*p for interaction* beta-blocker/perindopril 0.9 for no hypertension and 0.01 for hypertension).

When the analysis was performed using the current newer definition of hypertension (SBP/DBP ≥ 140/90), the treatment effect of the perindopril-based regimen on the primary endpoint was similar (beta-blocker stratum) with identical inferences (data not shown).

## Discussion

In this retrospective pooled analysis of three large perindopril outcome trials in patients at high cardiovascular risk, we found that adding a perindopril-based regimen to existing beta-blocker treatment reduced the risk of the primary composite endpoint of cardiovascular mortality, non-fatal MI, and stroke by 20% and secondary endpoints such as non-fatal MI by 23%, all-cause mortality by 22%, and cardiovascular mortality by 27% compared to placebo. These data suggest that the beneficial effects of perindopril treatment on patients’ survival are additive to the well-documented cardioprotective effects of beta-blockers [15–20]. Treatment with beta-blockers, for instance, has been shown to reduce mortality rates in patients with acute coronary syndrome [15, 21].

Perindopril outcome results are consistent with previously published data that have shown that perindopril treatment is associated with decreased rates of cardiovascular events and mortality in a broad range of patients with vascular disease [10–12, 22]. In particular, in the Van Vark et al. meta-analysis of 20 cardiovascular morbidity-mortality trials, perindopril data from the ASCOT-BPLA, ADVANCE, and HYVET trials drove the mortality benefits observed with ACE-inhibitors [22]. Direct and indirect comparisons between ACE-inhibitors have also confirmed the cardioprotective benefits of perindopril treatment [23, 24].

The cardioprotective and blood pressure reduction effects that we observed are likely due to complementary mechanisms of action. Treatment with a beta-blocker would be expected to prevent the release of renin that occurs as a feedback mechanism when perindopril inhibits the renin angiotensin aldosterone system [25]. Lowering renin levels could further reinforce the benefits of perindopril treatment by diminishing the risk of treatment failure. In addition, perindopril, when compared to other ACE-inhibitors, is a particularly potent inhibitor of bradykinin metabolism. Preserving bradykinin levels promotes vascular health by reducing blood pressure, oxidative stress, endothelial apoptosis, thrombin-induced platelet activation, inflammation, and fibrosis [25–32]. The effect of bradykinin preservation on vascular health, beyond blood pressure control, has yet to be understood in the context of beta-blockade. However, the data presented herein suggest that treating a patient with perindopril on a beta-blocker background supports the ability of perindopril to promote cardioprotection and vascular health given the additive risk reduction.

Regardless of what is known about mechanism of action, studies have shown that physicians often believe that the antihypertensive efficacy of ACE-inhibitors prescribed on top of a beta-blocker is not fully additive. Here, with this analysis, we were able to study whether such an effect exists. We show that at the end of the run-in period, treatment with perindopril decreased blood pressure regardless of background beta-blocker prescription and to the same degree in both groups (beta-blocker vs no beta-blocker). In other studies, such as the CONFIDENCE trial, adding perindopril to existing beta-blocker treatment led to significant decreases in blood pressure after 3 months [33].

Our data also illustrate the complexity of the molecular pathways at play. The beneficial effect of beta-blocker/perindopril treatment compared with beta-blocker/placebo treatment was significant for the primary endpoint and non-fatal MI in both hypertensive and non-hypertensive patients.

In real-life settings, the extent of blood pressure reduction and cardioprotective efficacy would most likely be marred by poor compliance. A 2008 Kaiser Permanente database analysis, for example, has shown that roughly 29% of patients with coronary artery disease were not compliant with their beta-blocker prescription and that non-compliance was associated with significantly higher risk of cardiovascular mortality (HR 1.53, 95% CI 1.16–2.01), all-cause mortality (HR 1.50, 95% CI 1.33–1.71), and revascularization procedures (HR 1.15, 95% CI 1.04–1.27) [34]. Other publications have estimated that 9% of cardiovascular disease events in Europe can be attributed to poor adherence to vascular medication and that good adherence to therapy decreases mortality risk [35, 36]. These data underscore the need to develop strategies in which patients can benefit from the full effect of treatment.

Among possible strategies, besides patient education by physicians and pharmacists, single-pill combinations are recommended by European guidelines to increase adherence [37]. They are associated with higher rates of adherence, better blood pressure control, and lower rates of cardiovascular events than free associations of components [38, 39]. Thus, a single-pill combination that would include perindopril and a beta-blocker would respond to a real need for single-pill combinations as a potential new option to reach further treatment benefits.

Beta-blocker type was not available in this analysis. The beta-blockers metoprolol and bisoprolol are the types most frequently prescribed in the world. Bisoprolol is a long-acting beta-blocker that is predominantly selective for β1-adrenoreceptors and that can be combined with perindopril in a single-pill formulation because, like perindopril, it has a 24-h duration of action [7, 40, 41]. Bisoprolol also benefits from a well-documented safety profile and has been classified as an essential drug for hypertension, angina, heart failure, and arrhythmia by the World Health Organization [42]. Its combination with perindopril is supported by data which have shown that bisoprolol/perindopril is safe and effective in blood pressure studies [32]. As the current analysis demonstrates a strong consistent treatment benefit of the ACE-inhibitor perindopril in patients with vascular disease on top of beta-blocker use and as this additive effect seems even more pronounced in patients with hypertension, the combination of perindopril and bisoprolol in a single-pill combination may offer treatment solutions to improve adherence.

Of note is the absence of a treatment benefit for the endpoints of stroke independent of the presence of hypertension or not, which is consistent with previous results. We did observe a mortality benefit of the combination perindopril on top of beta-blocker use overall and in hypertensive patients with a significant interaction effect between treatments used. In patients without hypertension, there was no effect on all-cause mortality.

### Limitations

Robustness of results in retrospective pooled analyses is often undermined by heterogeneity among trials. In order to reduce heterogeneity, we only included outcome trials with similar designs. All three studies, EUROPA, PROGRESS, and ADVANCE, were 4-year prospective, randomized, placebo-controlled trials that included patients with cardiovascular disease or patients at high risk of developing cardiovascular disease. Trial-specific definitions were used even though definitions and thresholds were not identical because statistical results have been shown to tolerate some heterogeneity in endpoint definitions [43]. In addition, we limited our trial selection to trials in which patients were randomized to a perindopril-based regimen. The ASCOT-BPLA trial, for instance, was not included because patients were randomized to an amlodipine-based regimen to which perindopril was added as needed [44]. The current analysis, thus, provides a unique and robust data set that assesses the treatment effect of perindopril in relation to background beta-blocker use. Regarding beta-blocker use, we only have data on baseline use. Doses of beta-blockers were not recorded. We, therefore, cannot evaluate how dose might have influenced outcome results. During the trials, standard of care was changing with the use of the combination of calcium antagonist and beta-blockers; such a time-trend may have confounded the effects. Additionally, during the course of the perindopril trials, there was the introduction of the long-acting metoprolol succinate in the presence of ongoing metoprolol tartrate usage No data on side-effects of beta-blockers or treatment effect heterogeneity were available related to beta-blocker use, which was described for perindopril use in these trials in detail previously [10–12, 45–47]. Finally, statistical power is lower in subgroup analyses.

## Conclusions

In this meta-analysis, a consistent treatment benefit was observed with a perindopril-based regimen on a background of beta-blocker use. This treatment benefit was most pronounced relatively in patients with hypertension, which could suggest an additive effect of both treatments.

## References

[1] Mancia G, Fagard R, Narkiewicz K, Redon J, Zanchetti A, Bohm M (2013). 2013 ESH/ESC guidelines for the management of arterial hypertension: the task force for the management of arterial hypertension of the European Society of Hypertension (ESH) and of the European Society of Cardiology (ESC). J Hypertens.

[2] Rosendorff C, Lackland DT, Allison M, Aronow WS, Black HR, Blumenthal RS (2015). Treatment of hypertension in patients with coronary artery disease: a scientific statement from the American Heart Association, American College of Cardiology, and American Society of Hypertension. J Am Coll Cardiol.

[3] Ponikowski P, Voors AA, Anker SD, Bueno H, Cleland JG, Coats AJ (2016). 2016 ESC guidelines for the diagnosis and treatment of acute and chronic heart failure: the task force for the diagnosis and treatment of acute and chronic heart failure of the European Society of Cardiology (ESC) developed with the special contribution of the Heart Failure Association (HFA) of the ESC. Eur Heart J.

[4] Thoenes M, Neuberger HR, Volpe M, Khan BV, Kirch W, Bohm M (2010). Antihypertensive drug therapy and blood pressure control in men and women: an international perspective. J Hum Hypertens.

[5] Kotseva K, Wood D, De Bacquer D, De Backer G, Ryden L, Jennings C (2016). EUROASPIRE IV: a European Society of Cardiology survey on the lifestyle, risk factor and therapeutic management of coronary patients from 24 European countries. Eur J Prev Cardiol.

[6] Maggioni AP, Anker SD, Dahlstrom U, Filippatos G, Ponikowski P, Zannad F (2013). Are hospitalized or ambulatory patients with heart failure treated in accordance with European Society of Cardiology guidelines? Evidence from 12,440 patients of the ESC Heart Failure Long-Term Registry. Eur J Heart Fail.

[7] Anderson PJ, Critchley JA, Tomlinson B, Resplandy G (1995). Comparison of the pharmacokinetics and pharmacodynamics of oral doses of perindopril in normotensive Chinese and Caucasian volunteers. Br J Clin Pharmacol.

[8] Nedogoda SV, Ledyaeva AA, Chumachok EV, Tsoma VV, Mazina G, Salasyuk AS (2013). Randomized trial of perindopril, enalapril, losartan and telmisartan in overweight or obese patients with hypertension. Clin Drug Investig.

[9] Taddei S (2015). RAS inhibitors’ dose-dependent efficacy: myth or reality?. Curr Med Res Opin.

[10] PROGRESS Collaborative group (2001). Randomised trial of a perindopril-based blood-pressure-lowering regimen among 6,105 individuals with previous stroke or transient ischaemic attack. Lancet.

[11] Fox KM (2003). EURopean trial on reduction of cardiac events with perindopril in stable coronary artery disease investigators. Efficacy of perindopril in reduction of cardiovascular events among patients with stable coronary artery disease: randomised, double-blind, placebo-controlled, multicentre trial (the EUROPA study). Lancet.

[12] Patel A, MacMahon S, Chalmers J, Neal B, Woodward M, Billot L (2007). Effects of a fixed combination of perindopril and indapamide on macrovascular and microvascular outcomes in patients with type 2 diabetes mellitus (the ADVANCE trial): a randomised controlled trial. Lancet.

[13] Brugts JJ, Ninomiya T, Boersma E, Remme WJ, Bertrand M, Ferrari R (2009). The consistency of the treatment effect of an ACE-inhibitor based treatment regimen in patients with vascular disease or high risk of vascular disease: a combined analysis of individual data of ADVANCE, EUROPA, and PROGRESS trials. Eur Heart J.

[14] Bertrand ME, Ferrari R, Remme WJ, Simoons ML, Fox KM (2015). Perindopril and beta-blocker for the prevention of cardiac events and mortality in stable coronary artery disease patients: a European trial on reduction of cardiac events with perindopril in stable coronary artery disease (EUROPA) subanalysis. Am Heart J.

[15] Raposeiras-Roubin S, Abu-Assi E, Redondo-Dieguez A, Gonzalez-Ferreiro R, Lopez-Lopez A, Bouzas-Cruz N (2015). Prognostic benefit of beta-blockers after acute coronary syndrome with preserved systolic function. Still relevant today?. Revista Espanola de Cardiologia.

[16] Law MR, Morris JK, Wald NJ (2009). Use of blood pressure lowering drugs in the prevention of cardiovascular disease: meta-analysis of 147 randomised trials in the context of expectations from prospective epidemiological studies. BMJ.

[17] Zhang H, Yuan X, Zhang H, Chen S, Zhao Y, Hua K (2015). Efficacy of long-term beta-blocker therapy for secondary prevention of long-term outcomes after coronary artery bypass grafting surgery. Circulation.

[18] Choo EH, Chang K, Ahn Y, Jeon DS, Lee JM, Kim DB (2014). Benefit of beta-blocker treatment for patients with acute myocardial infarction and preserved systolic function after percutaneous coronary intervention. Heart.

[19] Brugts JJ, den Uil CA, Danser AHJ (2009). The renin-angiotensin-aldosterone system: approaches to guide angiotensin-converting enzyme inhibition in patients with coronary artery disease. Cardiology.

[20] Brugts JJ, van Vark L, Akkerhuis M (2015). Impact of renin-angiotensin system inhibitors on mortality and major cardiovascular endpoints in hypertension: a number-needed-to-treat analysis. Int J Cardiol.

[21] Kernis SJ, Harjai KJ, Stone GW, Grines LL, Boura JA, O'Neill WW (2004). Does beta-blocker therapy improve clinical outcomes of acute myocardial infarction after successful primary angioplasty?. J Am Coll Cardiol.

[22] van Vark LC, Bertrand M, Akkerhuis KM, Brugts JJ, Fox K, Mourad JJ (2012). Angiotensin-converting enzyme inhibitors reduce mortality in hypertension: a meta-analysis of randomized clinical trials of renin-angiotensin-aldosterone system inhibitors involving 158,998 patients. Eur Heart J.

[23] Tsoi KK, Wong MC, Tam WW, Hirai HW, Lao XQ, Wang HH (2014). Cardiovascular mortality in hypertensive patients newly prescribed perindopril vs. lisinopril: a 5-year cohort study of 15,622 Chinese subjects. Int J Cardiol.

[24] Savarese G, Costanzo P, Cleland JG, Vassallo E, Ruggiero D, Rosano G (2013). A meta-analysis reporting effects of angiotensin-converting enzyme inhibitors and angiotensin receptor blockers in patients without heart failure. J Am Coll Cardiol.

[25] Gainer JV, Morrow JD, Loveland A, King DJ, Brown NJ (1998). Effect of bradykinin-receptor blockade on the response to angiotensin-converting-enzyme inhibitor in normotensive and hypertensive subjects. N Engl J Med.

[26] Ceconi C, Francolini G, Olivares A, Comini L, Bachetti T, Ferrari R (2007). Angiotensin-converting enzyme (ACE) inhibitors have different selectivity for bradykinin binding sites of human somatic ACE. Eur J Pharmacol.

[27] Oeseburg H, Iusuf D, van der Harst P, van Gilst WH, Henning RH, Roks AJ (2009). Bradykinin protects against oxidative stress-induced endothelial cell senescence. Hypertension.

[28] Murphey LJ, Malave HA, Petro J, Biaggioni I, Byrne DW, Vaughan DE (2006). Bradykinin and its metabolite bradykinin 1-5 inhibit thrombin-induced platelet aggregation in humans. J Pharmacol Exp Ther.

[29] Chao J, Li HJ, Yao YY, Shen B, Gao L, Bledsoe G (2007). Kinin infusion prevents renal inflammation, apoptosis, and fibrosis via inhibition of oxidative stress and mitogen-activated protein kinase activity. Hypertension.

[30] Ceconi C, Fox KM, Remme WJ, Simoons ML, Bertrand M, Parrinello G (2007). ACE inhibition with perindopril and endothelial function. Results of a substudy of the EUROPA study: PERTINENT. Cardiovasc Res.

[31] Ceconi C, Francolini G, Bastianon D, Gitti GL, Comini L, Ferrari R (2007). Differences in the effect of angiotensin-converting enzyme inhibitors on the rate of endothelial cell apoptosis: in vitro and in vivo studies. Cardiovasc Drugs Ther.

[32] Madej A, Buldak L, Basiak M, Szkrobka W, Dulawa A, Okopien B (2009). The effects of 1 month antihypertensive treatment with perindopril, bisoprolol or both on the ex vivo ability of monocytes to secrete inflammatory cytokines. Int J Clin Pharmacol Ther.

[33] Tsoukas G, Anand S, Yang K, Investigators C (2011). Dose-dependent antihypertensive efficacy and tolerability of perindopril in a large, observational, 12-week, general practice-based study. Am J Cardiovasc Drugs.

[34] Ho PM, Magid DJ, Shetterly SM, Olson KL, Maddox TM, Peterson PN (2008). Medication nonadherence is associated with a broad range of adverse outcomes in patients with coronary artery disease. Am Heart J.

[35] Chowdhury R, Khan H, Heydon E, Shroufi A, Fahimi S, Moore C (2013). Adherence to cardiovascular therapy: a meta-analysis of prevalence and clinical consequences. Eur Heart J.

[36] Simpson SH, Eurich DT, Majumdar SR, Padwal RS, Tsuyuki RT, Varney J (2006). A meta-analysis of the association between adherence to drug therapy and mortality. BMJ.

[37] Piepoli MF, Hoes AW, Agewall S, Albus C, Brotons C, Catapano AL (2016). 2016 European guidelines on cardiovascular disease prevention in clinical practice: the sixth joint task force of the European Society of Cardiology and other societies on cardiovascular disease prevention in clinical practice (constituted by representatives of 10 societies and by invited experts) developed with the special contribution of the European Association for Cardiovascular Prevention & Rehabilitation (EACPR). Eur Heart J.

[38] Egan BM, Bandyopadhyay D, Shaftman SR, Wagner CS, Zhao Y, Yu-Isenberg KS (2012). Initial monotherapy and combination therapy and hypertension control the first year. Hypertension.

[39] Belsey JD (2012). Optimizing adherence in hypertension: a comparison of outcomes and costs using single tablet regimens vs individual component regimens. J Med Econ.

[40] Davidov ME, Singh SP, Vlachakis ND, Blumenthal JB, Simon JS, Bryzinski BS (1994). Bisoprolol, a once-a-day beta-blocking agent for patients with mild to moderate hypertension. Clin Cardiol.

[41] Nuttall SL, Routledge HC, Kendall MJ (2003). A comparison of the beta1-selectivity of three beta1-selective beta-blockers. J Clin Pharm Ther.

[42] World Health Organization. WHO model list of essential medicines (April 2015). 2015.

[43] Early Breast Cancer Trialists’ Collaborative Group (1990). Treatment of early breast cancer. Worldwide evidence, 1985–1990.

[44] Dahlöf B, Sever PS, Poulter NR, Wedel H, Beevers DG, Caulfield M (2005). Prevention of cardiovascular events with an antihypertensive regimen of amlodipine adding perindopril as required versus atenolol adding bendroflumethiazide as required, in the Anglo-Scandinavian Cardiac Outcomes Trial-Blood Pressure Lowering Arm (ASCOT-BPLA): a multicentre randomised controlled trial. Lancet.

[45] Brugts JJ, de Maat MPM, Boersma E (2009). The rationale and design of the Perindopril Genetic Association Study (PERGENE): a pharmacogenetic analysis of angiotensin-converting enzyme inhibitor therapy in patients with stable coronary artery disease. Cardiovasc Drug Ther.

[46] Brugts JJ, Hisatomi A, Remme W (2014). The incidence and clinical predictors of ACE-inhibitor induced dry cough by perindopril in 27,492 patients with vascular disease. Int J Cardiol.

[47] Holmer SR, Hense HW, Danser AH, Mayer B, Riegger GA, Schunkert H (1998). Beta adrenergic blockers lower renin in patients treated with ACE inhibitors and diuretics. Heart.

